# Squeezed states generation by nonlinear plasmonic waveguides: a novel analysis including loss, phase mismatch and source depletion

**DOI:** 10.1038/s41598-023-27949-x

**Published:** 2023-01-19

**Authors:** Hamid Nadgaran, Mohammad Amin Izadi, Rahman Nouroozi

**Affiliations:** 1 Department of Physics, Shiraz University, Shiraz, 71454 Iran; 2 Department of Physics, Institute for Advanced Studies in Basic Sciences, Zanjan, 45137-66731 Iran

**Keywords:** Optics and photonics, Optical physics, Quantum optics

## Abstract

In this article, a full numerical method to study the squeezing procedure through second harmonic generation process is proposed. The method includes complex nonlinear coupling coefficient, phase mismatch, and pump depletion. Attention has been also paid to the effects of accumulated noises in this work. The final form of the numerical formula seems to be much simpler than the analytical solutions previously reported. The function of this numerical method shows that it works accurately for different mechanisms of squeezed state generations and does not suffer from instabilities usually encountered even for non-uniform, coarse steps. The proposed method is used to examine the squeezing procedure in an engineered nonlinear plasmonic waveguide. The results show that using the nonlinear plasmonic waveguide, it is possible to generate the squeezed states for the pump and the second harmonic modes with high efficiency in a propagation length as short as 2 mm which is much shorter than the needed length for the traditional nonlinear lithium niobate- based optical waveguides being of the order of 100 mm. This new method of squeezed states generation may find applications in optical communication with a noise level well below the standard quantum limit, in quantum teleportation, and in super sensitive interferometry.

## Introduction

Quantum optics is a flourishing branch of physics offering a unique path for the study of fundamental physics. Experimental and theoretical studies in this field are giving new and modern insights to our scientific view of the world^1–3^. Generation of light quantum states which have no counterparts in classical physics plays a central role in quantum optics. One of the fascinating quantum states studied by researchers in the field is squeezed states^4^. According to Heisenberg’s uncertainty principle, it is not possible to measure two conjugate quantities (or quadratures) with any arbitrary certainties simultaneously^5^. For coherent and vacuum states, the uncertainties in measuring the quadratures become equal and the Heisenberg’s uncertainty relation meets its minimum criterion^4^. This minimum criterion is related to so called the standard quantum limit (SQL)^6^. Surprisingly, the uncertainty in measuring one of the quadratures can be smaller than SQL for the squeezed states. Needless to say that stable squeezed states has opened a new era in applications, such as in optical communication with a noise level well below SQL^7,8^, quantum teleportation^9,10^ and super sensitive interferometry^11–13^.

The generation of squeezed states seemed almost impossible around 1985^14^. The first signs of the squeezing states generation were reported by Slusher et al.^15^. They used an atomic vapor of sodium atoms as the suitable environment of four-wave-mixing to generate the squeezed states. Their experimental set up occupied a whole optical bench and therefore, the generated squeezed states could not be directly used in optical circuitry. Thereafter, an experimental method using second order nonlinear crystals was introduced by Wu who proved that the generation of squeezed state can be very much feasible using nonlinear crystals and waveguides and their related compactness^16^. This experiment proved that second order nonlinear crystals and waveguides are suitable and compact tools to generate the squeezed states. However, because of smallness of the nonlinear coefficient of the traditional nonlinear optical crystals, the generation rate of the squeezed states was not high^17^.

While the traditional field of quantum optics/photonics utilize dielectric structures like linear and nonlinear optical waveguides and fibers, the field of quantum plasmonics dealing with quantum properties of light and its interaction with metallic materials at the nano-scale is a rapidly growing field^18–25^. In plasmonic systems, surface plasmon polariton (SPP) modes are coupled excitations of electromagnetic waves to the electron charge density mode on metal-dielectric interfaces. These modes enable the confinement of light in nano-scale sizes that could be well below the diffraction limit. Therefore, plasmonic structures pave the way to miniaturize the foot-print of optical circuits and enhance the nonlinear coupling coefficients^26,27^. Nonlinear optical waveguides loaded with metals is regarded as SPP based second order nonlinear media which can be used to generate squeezed states. Due to much higher nonlinear coupling coefficients achieved in these systems compared to that of the traditional nonlinear dielectric waveguides, SPP based nonlinear waveguides can well enhance the performance of squeezed states^28^. One can expect that SPP based squeezed states would take this science to the realm of nano optics^29^.

Although, there are lots of theoretical works in the literature for analytical formulating of the squeezing procedure through the traditional second order nonlinear optical waveguides^17,30,31^, their direct application to plasmonic waveguides for squeezed state generation cannot be easily accomplished. The main problems include various approximations and simplifications usually made. For example, the nonlinear coupling coefficient was considered real, while this parameter is essentially a complex number due to phase difference between the interacting modes^6,32^. This sort of nonlinear phase mismatching is so profound in nonlinear plasmonic waveguides and researchers usually work with quasi phase matching (QPM) techniques^33^. In essence therefore, residual nonlinear phase mismatching between the interacting modes cannot be ignored in order to avoid performance reduction of the squeezed states generation process. Furthermore, in some studies, pump depletion was ignored^17^, which cannot be true in general, since in nonlinear interaction, the pump dissipates its energy to other modes. Pump depletion becomes more important in plasmonic waveguides where strong nonlinear interactions are present^32,34,35^. Having named the above various simplifications, one must pay attention to effects of loss mechanism, since the accumulated noises resulted from the loss mechanisms lead to de-squeezing of the quadratures^1,36,37^. This issue will become more critical in squeezing procedures using nonlinear plasmonic waveguides, where the intrinsic loss of SPP modes are strong^26,28^. Therefore, construction a simple but intelligent numerical method for squeezing procedure which relaxes various above mentioned simplifications has a great motivation.

This article is organized as follows:

- In “Methods” section, our intelligent simple numerical method is introduced by describing the squeezing procedure through second harmonic generation (SHG). Also, the method of beam splitter for including the loss mechanism will be given. The proposed engineered nonlinear plasmonic waveguide used to generate the squeezed states is then introduced.
- In “Results and discussion” section, the accuracy of the proposed method will be discussed and the generation of the squeezed states through the nonlinear plasmonic waveguides will be presented. The squeezing performance and the superiority of the nonlinear plasmonic waveguide over other conventional methods in this respect will be addressed
- The article will then be concluded by analysis of the results.

## Methods

The prime aim of this section is to present a numerical method to calculate the squeezing procedure of the quantum optical quadratures when second order nonlinear optical waveguides are used. Equation (1) show the classical SHG process in a nonlinear waveguide:

1 $$\begin{aligned} \begin{aligned} \frac{\textrm{d}A_{\omega }}{\textrm{d}x}&= j\kappa ^{*}B_{2\omega }A_{\omega }^{*}\exp (j\Delta \beta x)-f_p A_{\omega },\\ \frac{\textrm{d}B_{2\omega }}{\textrm{d}x}&= j\kappa A_{\omega }^{2}\exp (-j\Delta \beta x)-f_{sh} B_{2\omega }, \end{aligned} \end{aligned}$$

where $A_{\omega }$, $B_{2\omega }$ and $\kappa$ are the amplitude of the electric field of the pump mode, second harmonic (SH) mode and the complex nonlinear coupling coefficient, respectively^32,34^. Also, $\Delta \beta$ is the nonlinear phase mismatch between the interacting modes^32,34^. Furthermore, $f_p$ and $f_{sh}$ represent the attenuation coefficients of the pump and SH amplitudes, respectively. To achieve the quantum mechanical counterpart, classical electric field amplitudes should be replaced with electric field operators via^4^:

2 $$\begin{aligned} \begin{aligned} A_{\omega }&= \sqrt{\frac{\hbar \omega }{\varepsilon _0 L}}\hat{a}, \\ B_{2\omega }&= \sqrt{\frac{2 \hbar \omega }{\varepsilon _0 L}}\hat{b}. \end{aligned} \end{aligned}$$

Here, $\omega$ and *L* are the fundamental angular frequency and the waveguide length, respectively. Furthermore, $\hat{a}$ and $\hat{b}$ represent the quantum mechanical operators for the pump and SH electric field amplitudes, respectively. If Eq. (2) are substituted in Eq. (1), the nonlinear coupled equations describing the SHG in the quantum optics picture will be achieved:

3 $$\begin{aligned} \begin{aligned} \frac{{\textrm{d}}{\hat{a}}}{\textrm{d}x}& = j\kappa ^{*}_q {\hat{b}}{\hat{a}}^{\dagger } \exp (j\Delta \beta x)-f_p {\hat{a}},\\ \frac{{\textrm{d}}{\hat{b}}}{\textrm{d}x}& = j\frac{\kappa _q}{2} {\hat{a}}^{2}\exp (-j\Delta \beta x)-f_{sh}{\hat{b}}. \end{aligned} \end{aligned}$$

In Eqs. (3), $\kappa _q=\sqrt{\frac{2 \hbar \omega }{\epsilon _0 L}}\kappa$ is the quantum nonlinear coupling coefficient. Obviously, each electric field operator can be shown by its expectation value ($\left\langle \hat{a}\right\rangle$) and difference operator ($\Delta \hat{a}$):

4 $$\begin{aligned} \begin{aligned} \hat{a}&=\left\langle \hat{a}\right\rangle +\Delta \hat{a},\\ \hat{b}&=\left\langle \hat{b}\right\rangle +\Delta \hat{b}. \end{aligned} \end{aligned}$$

The uncertainty of operator $\hat{a}$ can be calculated via the square root of its variance:

5 $$\begin{aligned} \sigma _a=\sqrt{\left\langle a^2\right\rangle -\left\langle a\right\rangle ^2}. \end{aligned}$$

Substituting Eq. (4) in Eq. (3), two sets of coupled equations for the expectation values and difference operators up to zeroth order approximation can be given as follows:

6 $$\begin{aligned} \begin{aligned} \frac{{\textrm{d}}\left\langle \hat{a}\right\rangle }{{\textrm{d}}x}&= j\kappa ^{*}_q \left\langle \hat{b}\right\rangle \left\langle \hat{a}\right\rangle ^{*} \exp (j\Delta \beta x)-f_p \left\langle \hat{a}\right\rangle ,\\ \frac{{\textrm{d}}\left\langle \hat{b}\right\rangle }{{\textrm{d}}x}&= j\kappa _q \left\langle \hat{a}\right\rangle ^{2}\exp (-j\Delta \beta x)-f_{sh}\left\langle \hat{b}\right\rangle , \end{aligned} \end{aligned}$$

and

7 $$\begin{aligned} \begin{aligned} \frac{{\textrm{d}}\Delta \hat{a}}{{\textrm{d}}x}&= j\kappa ^{*}_q \left\{ \left\langle \hat{b}\right\rangle \Delta \hat{a}^{\dagger }+ \left\langle \hat{a}\right\rangle ^{*} \Delta \hat{b} \right\} \exp (j\Delta \beta x), \\ \frac{{\textrm{d}}\Delta \hat{b}}{{\textrm{d}}x}&= j\kappa _q \left\{ \left\langle \hat{a}\right\rangle \Delta \hat{a} + 0 \Delta \hat{b} \right\} \exp (-j\Delta \beta x). \end{aligned} \end{aligned}$$

In writing Eq. (7), term $\left( \Delta \hat{a}^{\dagger } \Delta \hat{b}\right)$ is omitted to obtain a linearized form^31^. Although, this can potentially be a source of inaccuracy of the results, its consequences can be minimized using very tiny steps in numerical calculations. Moreover, the effects of loss are not considered in Eq. (7) since it is a complicated procedure and needs a quantum mechanical considerations. The process of including loss mechanisms in the behavior of the difference operators will be discussed soon. Equation (6) are nonlinear coupled equations with respect to the expectation values. On the other hand, Eq. (7) are linear with respect to the difference operators. To solve Eq. (6), the well-known method of Runge–Kutta can be used^38^. Here, items for complexity for pump depletion, complex nonlinear coupling coefficient and nonlinear phase mismatch can be taken into account. This is in sharp contrast to analytical approaches, where for simplicity, do not consider any of the above mentioned items.

Now, a suitable numerical solution for Eq. (7) should be provided. To begin with, Eq. (7) are rewritten along with their Hermitian conjugates:

8 $$\begin{aligned} \begin{aligned} \frac{{\textrm{d}}\Delta \hat{a}}{{\textrm{d}}x}&= j\kappa ^{*}_q \left\{ \left\langle \hat{b}\right\rangle \Delta \hat{a}^{\dagger }+ \left\langle \hat{a}\right\rangle ^{*} \Delta \hat{b} \right\} \exp (j\Delta \beta x), \\ \frac{{\textrm{d}}\Delta \hat{a}^{\dagger }}{{\textrm{d}}x}&= -j\kappa _q \left\{ \left\langle \hat{b}\right\rangle ^{*} \Delta \hat{a}+ \left\langle \hat{a}\right\rangle \Delta \hat{b}^{\dagger } \right\} \exp (-j\Delta \beta x), \\ \frac{{\textrm{d}}\Delta \hat{b}}{{\textrm{d}}x}&= j\kappa _q \left\{ \left\langle \hat{a}\right\rangle \Delta \hat{a} + 0 \Delta \hat{b} \right\} \exp (-j\Delta \beta x),\\ \frac{{\textrm{d}}\Delta \hat{b}^{\dagger }}{{\textrm{d}}x}&= -j\kappa _q^{*} \left\{ \left\langle \hat{a}\right\rangle ^{*} \Delta \hat{a}^{\dagger } + 0 \Delta \hat{b}^{\dagger } \right\} \exp (j\Delta \beta x). \end{aligned} \end{aligned}$$

These equations are linear and they can be cast in a matrix form:

9 $$\begin{aligned} \frac{\textrm{d}}{\textrm{d}x} \begin{pmatrix} \Delta \hat{a}\\ \Delta \hat{a}^{\dagger }\\ \Delta \hat{b}\\ \Delta \hat{b}^{\dagger } \end{pmatrix}= \begin{pmatrix} 0&{}j\kappa ^{*}_q\left\langle \hat{b}\right\rangle \exp (j\Delta \beta x)&{}j\kappa ^{*}_q\left\langle \hat{a}\right\rangle ^{*}\exp (j\Delta \beta x)&{}0\\ -j\kappa _q\left\langle \hat{b}\right\rangle ^{*}\exp (-j\Delta \beta x)&{}0&{}0&{}-j\kappa _q\left\langle \hat{a}\right\rangle \exp (-j\Delta \beta x)\\ j\kappa _q\left\langle \hat{a}\right\rangle \exp (-j\Delta \beta x)&{}0&{}0&{}0\\ 0&{}-j\kappa _q^{*}\left\langle \hat{a}\right\rangle ^{*}\exp (j\Delta \beta x)&{}0&{}0 \end{pmatrix} \begin{pmatrix} \Delta \hat{a}\\ \Delta \hat{a}^{\dagger }\\ \Delta \hat{b}\\ \Delta \hat{b}^{\dagger } \end{pmatrix} \end{aligned}$$

Since $\frac{\textrm{d}T}{\textrm{d}x}\approx \frac{T\left( h \right) -T\left( 0 \right) }{h}$, using forward approximation of derivatives for a tiny step of length *h*, we have:

10 $$\begin{aligned} \frac{\textrm{d}}{\textrm{d}x} \begin{pmatrix} \Delta \hat{a}\left( x \right) \\ \Delta \hat{a}^{\dagger }\left( x \right) \\ \Delta \hat{b}\left( x \right) \\ \Delta \hat{b}^{\dagger }\left( x \right) \end{pmatrix}\approx \frac{1}{h}\left[ \begin{pmatrix} \Delta \hat{a}\left( h \right) \\ \Delta \hat{a}^{\dagger }\left( h \right) \\ \Delta \hat{b}\left( h \right) \\ \Delta \hat{b}^{\dagger }\left( h \right) \end{pmatrix}-\begin{pmatrix} \Delta \hat{a}\left( 0 \right) \\ \Delta \hat{a}^{\dagger }\left( 0 \right) \\ \Delta \hat{b}\left( 0 \right) \\ \Delta \hat{b}^{\dagger }\left( 0 \right) \end{pmatrix} \right] \end{aligned}$$

If $\left[ \Delta \hat{a}\left( h \right) \right]$ and $\left[ \Delta \hat{a}\left( 0 \right) \right]$ represent the total matrix of the difference operators at step *h* and at the beginning point respectively, we will have:

11 $$\begin{aligned} \text {U}\left[ \Delta \hat{a}\left( h \right) \right] = \left[ \Delta \hat{a}\left( 0\right) \right] , \end{aligned}$$

where U is:

12 $$\begin{aligned} \begin{pmatrix} 1&{}-j\kappa ^{*}_q\left\langle \hat{b}\right\rangle \exp (j\Delta \beta x)&{}-j\kappa ^{*}_q\left\langle \hat{a}\right\rangle ^{*}\exp (j\Delta \beta x)&{}0\\ j\kappa _q\left\langle \hat{b}\right\rangle ^{*}\exp (-j\Delta \beta x)&{}1&{}0&{}j\kappa _q\left\langle \hat{a}\right\rangle \exp (-j\Delta \beta x)\\ -j\kappa _q\left\langle \hat{a}\right\rangle \exp (-j\Delta \beta x)&{}0&{}1&{}0\\ 0&{}j\kappa _q^{*}\left\langle \hat{a}\right\rangle ^{*}\exp (j\Delta \beta x)&{}0&{}1. \end{pmatrix} \end{aligned}$$

Therefore, it is straightforward to show that the difference operators for a tiny step *h* just after the beginning point can be related to the difference operators at the entrance point as what follows:

13 $$\begin{aligned} \left[ \Delta \hat{a}\left( h \right) \right] = \text {U}^{-1} \left[ \Delta \hat{a}\left( 0\right) \right] . \end{aligned}$$

The covariance matrix method can be used to calculate the evolution of the uncertainties of the quadratures^6^. The covariance matrix at step *h* and at the beginning point are defined as:

14 $$\begin{aligned} \begin{aligned} \text {V}\left( h \right)&=\left\langle \left[ \Delta \hat{a}\left( h \right) \right] \left[ \Delta \hat{a}\left( h \right) \right] ^{\dagger } \right\rangle , \\ \text {V}\left( 0 \right)&=\left\langle \left[ \Delta \hat{a}\left( 0 \right) \right] \left[ \Delta \hat{a}\left( 0 \right) \right] ^{\dagger } \right\rangle . \end{aligned} \end{aligned}$$

In SHG process, the laser beam at angular frequency $\omega$ entering into the nonlinear waveguide is in the $\vert {\alpha } \rangle$ state, while the initial state of the light at angular frequency $2\omega$ is vacuum $(\vert {0} \rangle )$^31^. Therefore, the covariance matrix at the beginning point is^6^:

15 $$\begin{aligned} \text {V}\left( 0 \right) = \begin{pmatrix} 1&{}0&{}0&{}0\\ 0&{}0&{}0&{}0\\ 0&{}0&{}1&{}0\\ 0&{}0&{}0&{}0 \end{pmatrix}. \end{aligned}$$

Using equations 14, we have:

16 $$\begin{aligned} \text {V}\left( h\right) =\left\langle \left[ \Delta \hat{a}\left( h \right) \right] \left[ \Delta \hat{a}\left( h \right) \right] ^{\dagger } \right\rangle = \left\langle \text {M}\left[ \Delta \hat{a}\left( 0 \right) \right] \left[ \Delta \hat{a}\left( 0 \right) \right] ^{\dagger }\text {M}^{\dagger }\right\rangle =\text {M}\text {V}\left( 0 \right) \text {M}^{\dagger }, \end{aligned}$$

where M is $\text {U}^{-1}$. Using this method, it is possible to calculate the covariance matrix at the following steps along the optical waveguide via iteration method:

17 $$\begin{aligned} \text {V}_{N+1}=\text {M}\text {V}_{N}\text {M}^{\dagger }. \end{aligned}$$

The squeezing factor for the quadratures of the pump $\left( \frac{\hat{a}+\hat{a}^{\dagger }}{2}\right)$ and SH $\left( \frac{\hat{b}+\hat{b}^{\dagger }}{2}\right)$ modes can be calculated via the following formula, respectively^6^:

18 $$\begin{aligned} S_p=\frac{1}{4}\left\{ v_{11}+v_{22}-2\left| v_{12}\right| \right\} , \end{aligned}$$

and

19 $$\begin{aligned} S_{sh}=\frac{1}{4}\left\{ v_{33}+v_{44}-2\left| v_{34}\right| \right\} , \end{aligned}$$

where $v_{ij}$ are the elements of the covariance matrix V.

**Figure 1:**
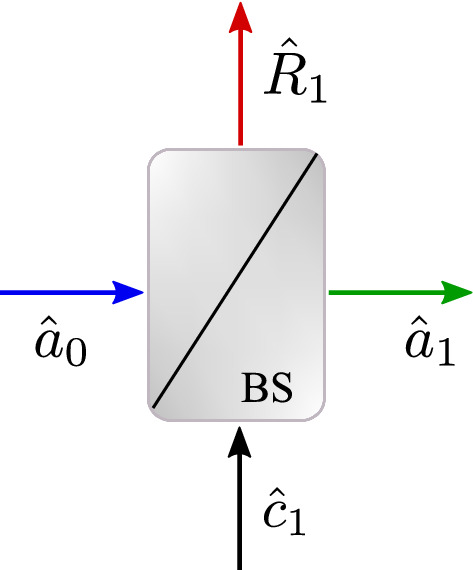
Each loss center can be simulated with a BS where operator $\hat{a}_{0}$ enters into the BS and mixes with noise $\hat{c_1}$. operator $\hat{a}_{1}$ goes out of it and the BS reflects operator $\hat{R_1}$.

Although many experiments of quantum optics are performed in vacuum, a nonlinear optical waveguide was taken as a medium for squeezing of quadratures. Therefore, the effect of loss and the related added noises should be included. Here, we describe the method of beam splitter (BS) introduced by Loudon^1,36^. Figure 1 illustrates a typical loss center.

As one can see, operator $\hat{a}_{0}$ enters BS and operator $\hat{a}_{1}$ goes out of it. In the BS, operator $\hat{a}_{0}$ combines with a noise operator $\hat{c_1}$. Also the BS reflects operator $\hat{R_1}$. If *t* and *r* are the transmittance and reflectance coefficients of the BS, respectively, we have:

20 $$\begin{aligned} \left| t\right| ^2+\left| r\right| ^2=1. \end{aligned}$$

According to the procedure, operator $\hat{a}_{1}$ is:

21 $$\begin{aligned} \hat{a}_{1}=t\hat{a}_{0}+r \hat{c}_{1}. \end{aligned}$$

It should be noted that at the beginning of the SHG process, there are two different entrance modes i.e., the pump mode at frequency $\omega$ and the vacuum state for the SH mode. Therefore, at each loss center, two noises affecting the pump and the SH modes should be included. As a result, the transferred difference operators for the pump and the SH modes can be achieved from each loss center (BS) as below:

22 $$\begin{aligned} \begin{pmatrix} \Delta \hat{a}_{N}\\ \Delta \hat{a}^{\dagger }_{N}\\ \Delta \hat{b}_{N}\\ \Delta \hat{b}^{\dagger }_{N} \end{pmatrix}= \text {T} \begin{pmatrix} \Delta \hat{a}_{N-1}\\ \Delta \hat{a}^{\dagger }_{N-1}\\ \Delta \hat{b}_{N-1}\\ \Delta \hat{b}^{\dagger }_{N-1} \end{pmatrix}+ \text {R} \begin{pmatrix} \Delta \hat{c}_{N}\\ \Delta \hat{c}^{\dagger }_{N}\\ \Delta \hat{g}_{N}\\ \Delta \hat{g}^{\dagger }_{N}, \end{pmatrix} \end{aligned}$$

where $\hat{c}$ and $\hat{g}$ denote the noise operators which combine with the pump and with the SH modes at each step, respectively. If the amplitude of electric field for the pump and SH modes attenuate as $\exp (-f_{p}x)$ and $\exp (-f_{sh} x)$, matrix T will be:

23 $$\begin{aligned} \begin{pmatrix} t_p&{}0&{}0&{}0\\ 0&{}t_p&{}0&{}0\\ 0&{}0&{}t_{sh}&{}0\\ 0&{}0&{}0&{}t_{sh} \end{pmatrix} \end{aligned}$$

where $t_p=\exp (-f_{p}x)$ and $t_{sh}=\exp (-f_{sh}x)$. Furthermore, the matrix R can be cast as what follows:

24 $$\begin{aligned} \begin{pmatrix} r_p&{}0&{}0&{}0\\ 0&{}r_p&{}0&{}0\\ 0&{}0&{}r_{sh}&{}0\\ 0&{}0&{}0&{}r_{sh} \end{pmatrix} \end{aligned}$$

In this matrix, using Eq. (20), we have: $r_p=\sqrt{1-t_p^2}$ and $r_{sh}=\sqrt{1-t_{sh}^2}$. This is where the strength of the numerical method of calculating the squeezing process in the presence of loss mechanisms is seen. To use this numerical method, it is enough to divide the length of the waveguide into very small pieces (steps of length *h*). Each step contains two separate levels:

- At first level, no loss mechanism is considered, using the numerical method described above, the squeezing procedure is calculated for this step and the output is saved.
- For the second level, the output of the previous level is considered as the input and using the BS explained earlier, the effects of the loss mechanism and the related noises for the pump and for the SH modes are calculated. The outputs of this level are considered as the input for the next step.

Combining these two levels, for $\text {V}_{N}$, we will have:

25 $$\begin{aligned} \text {V}_{N}=\text {TMV}_{N-1}\text {M}^{\dagger }\text {T}^{\dagger }+\text {R}\begin{pmatrix} 1&{}0&{}0&{}0\\ 0&{}0&{}0&{}0\\ 0&{}0&{}1&{}0\\ 0&{}0&{}0&{}0 \end{pmatrix}\text {R}^{\dagger }. \end{aligned}$$

Equation (25) contains the covariance matrix for the noises. It should be noted that any covariance matrix like $\left\langle \text {TM}\left[ \Delta a_{N-1} \right] \left[ \Delta c_N\right] ^{\dagger } \text {R}^{\dagger } \right\rangle$ which includes the product of operators $\Delta a_{N-1}$ and $\Delta c_{N}$ is zero because, there is no correlation between the newly arrived noises and the previous difference operators (they are independent)^31^. As one can see, this final numerical expression is much simpler than other analytical relations^39^ and contains various types of previous items mentioned above.

**Figure 2:**
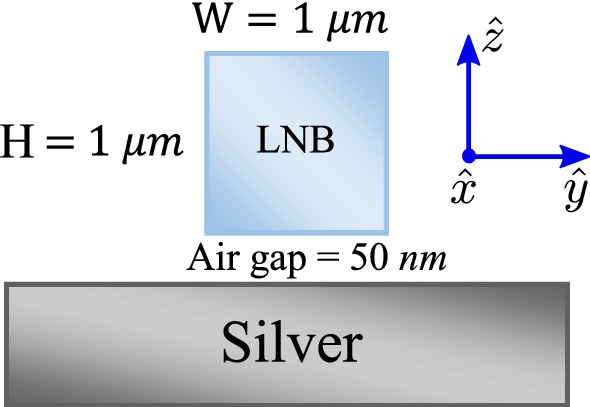
The cross section ($\hat{y}$-$\hat{z}$) of the hybrid nonlinear plasmonic waveguide. An air gap is embedded between the LNB and silver substrate. $\hat{x}$ is the propagation direction and $\hat{z}$ is along the optical axis of the LNB layer.

Finally, the structure of the proposed SPP nonlinear waveguide is introduced here. Figure 2 illustrates the cross section of the hybrid plasmonic waveguide where a tiny air gap between the LNB layer and the silver substrate is embedded^27^. $\hat{x}$ and $\hat{z}$ are the propagation direction and the direction of the optical axis of LNB crystal, respectively.

The physical dimensions of the hybrid waveguide are shown in Fig. 2. The optical properties of this hybrid waveguide can be achieved using finite difference method^40^.

## Results and discussion

**Figure 3:**
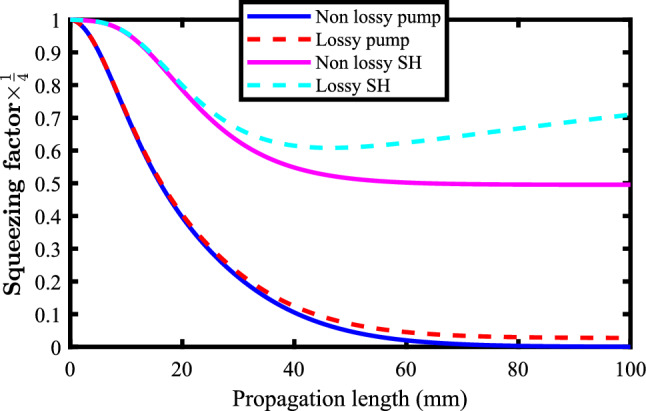
Quadratures squeezing in Ti:LNB waveguide for the SHG process. Without the loss mechanism, the pump mode (blue-solid line) squeezes unboundedly, while the SH mode (magenta-solid line) is squeezed to half its initial value. When the loss mechanism is included, the squeezing rate of the pump mode (red-dashed line) is limited and the SH mode (cyan-dashed line) de-squeezes due to accumulated noises.

To begin with, the accuracy of the numerical method should be examined and be compared with the well-established analytical results^17^ for the squeezing procedure through SHG. To do this, a traditional quasi phase matched Ti:LNB nonlinear optical waveguide with $\kappa =63 \frac{1}{\sqrt{{W}}{m}}$ was considered. Also, the attenuation coefficients for the pump at $\lambda _p={1550} \;{\text {nm}}$ and for the SH modes at $\lambda _{sh}={775}\;{\text {nm}}$ are $f_p=1.7269 \frac{1}{{ m}}$ and $f_{sh}=3.4539 \frac{1}{{ m}}$, respectively^41^. Figure 3 shows the squeezing procedures in the the SHG process for the pump and SH modes in the absence and in the presence of the loss mechanisms. The initial pump power ($\text {P}_{0}$) was 1*W*.

**Figure 4:**
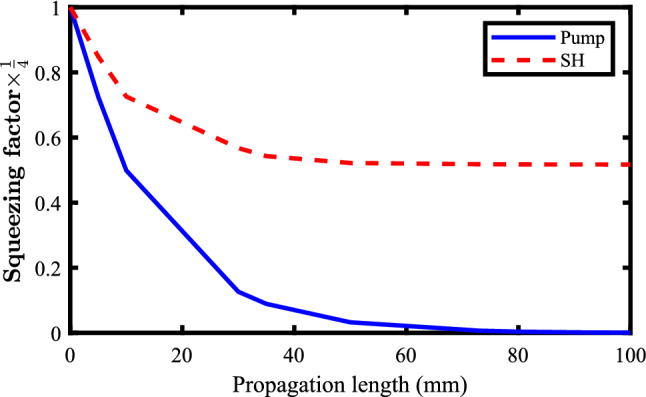
Quadrature squeezing for the pump (blue-solid line) and the SH mode (dashed-solid line) when just 10 non-uniform coarse steps are used to examine the stability of the method.

From the figure, it can be seen that in the absence of the loss mechanism, the pump mode is unboundedly squeezed to zero, while the SH mode is squeezed to half its initial value. These results coincide with the previous works reported in Ref.^17^ indicating the feasibility and reliability of the proposed method. The accuracy of the method was tested using different squeezing scenarios for different types of second order nonlinear phenomena. The results are in good agreement with the published works. Moreover, our numerical method was found to be stable even for non-uniform coarse steps. Figure 4 shows the pump and the SH modes squeezing when just 10 non-uniform coarse steps are considered for 100 mm. As it can be seen, using nonuniform coarse mesh does not affect the accuracy and the stability of the method.

In this respect it is worth to mention that the main source of instability of the proposed method is using ultra tiny steps that leads to accumulated arithmetic errors usually encounters in computing. Therefore, ultra tiny steps should be avoided. In this article, 1000 steps were used to achieve the results. The infinite squeezing of the pump mode to zero is just a mathematically affordable solution without any physical sense, because it does need infinite energy and infinite photon number (see equation 2.143 in Ref.^4^). However, one notes that in the presence of loss mechanism, the pump mode is squeezed to a finite value being greater than zero. This is the result of the accumulated noises of the medium environment of the optical waveguide. Also as one can note, these noises limits the efficiency of the generation of the squeezed states. This means, as the SH frequency becomes doubled through SHG process, its attenuation is stronger and as a result, the accumulated noises will act in a much stronger manner than that of the pump mode. Accordingly, the SH mode is squeezed and the rate of the squeezing decreases in the presence of loss leading to SH mode de-squeezing. Finally, the results predict the length of the LNB waveguide to be about 100 mm for final squeezing rate of the pump mode.

**Figure 5:**
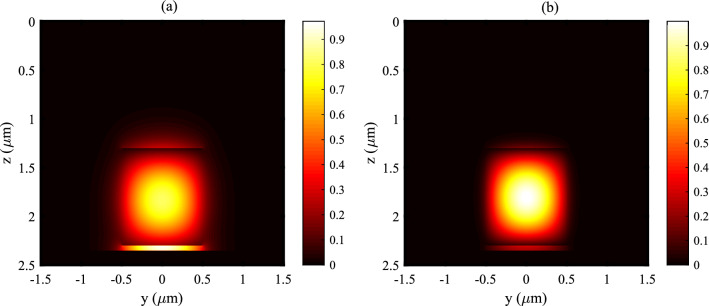
The absolute value of electric field along the $\hat{z}$ direction for the TM plasmonic based hybrid mode at $\lambda _p={1550} \; {\text {nm}}$ (**a**) and the TM true guided mode at $\lambda _{sh}={775} \; {\text {nm}}$ (**b**).

Finite difference (FD) method was employed for calculation of the optical properties of the nonlinear plasmonic waveguide depicted in Fig. 2. TThe waveguide is engineered in such a way that it guides two different transverse magnetic (TM) modes. Figure 5a shows the absolute value of electric field along the $\hat{z}$ direction for the plasmonic based hybrid mode at $\lambda _p={1550} \; {\text {nm}}$.

As it can be seen, this mode is a hybrid combination of the SPP mode attaching the surface of the silver layer and the guided mode of the LNB waveguide. Figure 5b illustrates the absolute value of electric field along the $\hat{z}$ direction for the guided mode at $\lambda _{sh}={775}\;{\text {nm}}$. From the figure, it can be noted that the main part of the electric field at$\lambda _{sh}={775}\;{\text {nm}}$ locates in the LNB which can be trusted as an almost a true guided mode^27^. QPM approach was utilized to ignore any absolute nonlinear phase mismatch between the interacting modes. It is worth pointing out that, due to the large overlap between these two modes in a tiny cross section of the LNB layer, the nonlinear coupling coefficient is much greater than that of the traditional LNB optical waveguide. The calculation shows that for the plasmonic nonlinear waveguide, $\kappa =713.15+2.4 i~\frac{1}{\sqrt{{{ Wm}}}}$. As a result, the nonlinear coupling coefficient of the plasmonic waveguide is about 11 times that of the traditional LNB optical waveguide. The propagation constants of the SPP and the guided modes are $\beta _p=1.9364+3.9\times 10^{-5}~\frac{1}{{ m}}$ and $\beta _{sh}=2.12+1.1\times 10^{-6}~\frac{1}{{ m}}$, respectively.

**Figure 6:**
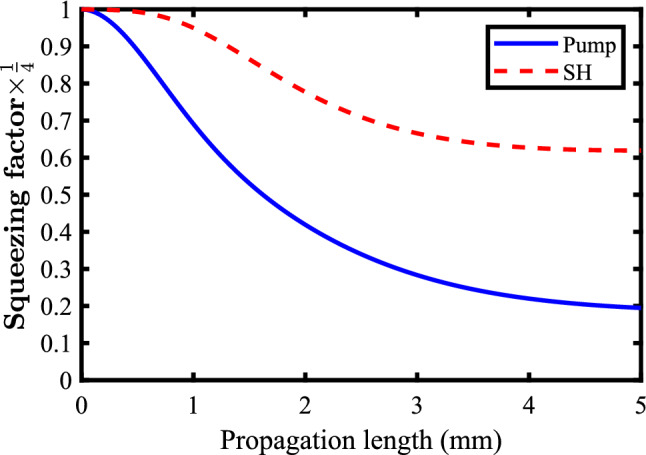
Pump (blue-solid line) and the SH mode (red-dashed line) squeezing through the nonlinear plasmonic waveguide proposed in Fig. 2. Here, the initial pump power $(\text {P}_{0})$ is 1*W*.

Figure 6 shows the squeezing procedure in plasmonic waveguide for the SHG process. The initial pump power ($\text {P}_{0}$) which excites the SPP mode is 1*W*. The results proposes the superiority of the performance of nonlinear plasmonic waveguide over its traditional Ti:LNB nonlinear waveguide counterparts. This can be more welcome when this work is pleased to report that the nonlinear plasmonic waveguide squeezes the pump and the SH modes in a propagation length about ${5}\;{\text {mm}}$ which is much shorter than the required propagation length of ${100}\;{\text {mm}}$ for squeezing through traditional LNB waveguides.

The final rate of pump mode squeezing is however, lower than that of the traditional nonlinear Ti:LNB waveguide (see Fig. 8). Accumulated noises of the plasmonic mode is to blame for this lower pump mode squeezing rate.

**Figure 7:**
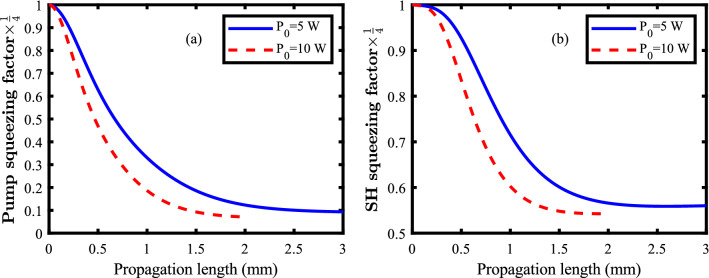
Squeezing of the pump mode (**a**) and the SH mode (**b**) for different values of the initial pump power ($\text {P}_{0}=5{{ W}}~\text {and}~10{{ W}}$).

**Figure 8:**
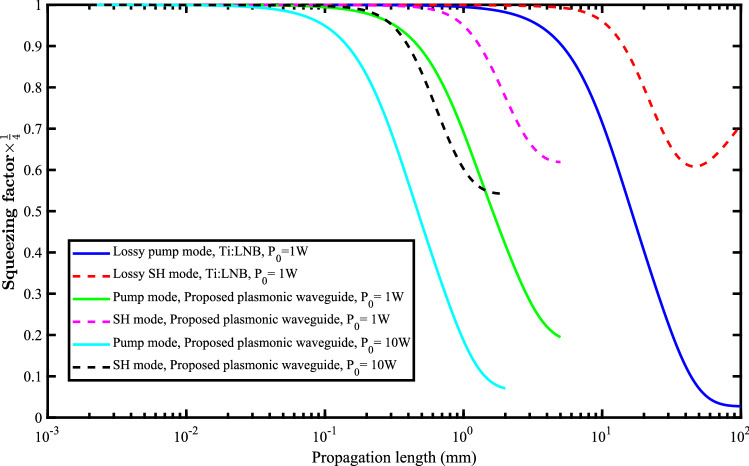
Squeezing of the pump mode (blue-solid line) and SH mode (red-dashed line) in the traditional Ti:LNB waveguide ($\text {P}_{0}= 1{{ W}}$). Squeezing of the pump mode (green-solid line) and the SH mode (magenta-dashed line) in the proposed plasmonic waveguide for $\text {P}_{0}= 1{{ W}}$. Squeezing of the pump mode (cyan-solid line) and the SH mode (black-dashed line) in the proposed plasmonic waveguide for $\text {P}_{0}= 10{{ W}}$. The log-scale is used for the horizontal axis.

The effects of higher values of the initial pump power ($\text {P}_{0}=5~\text {and}~10{{ W}}$) on the squeezing efficiency are shown in Fig. 7 for the pump mode (a) and the SH mode (b). This is done to enhance the efficiency of the proposed nonlinear plasmonic waveguide. It should be noted, that although sub-watt lasers are usually used in quantum optics experiments, as the generation of the squeezed states are a multi-photon process, use of multi-watt lasers ($\sim 10{{ W}}$) is also reported quite well in the literature^30,39,42^. However, elegant and careful experimental set-ups are needed to avoid heat and any undesired linear and nonlinear effects that might mask the desired squeezing. Proper cooling is also reported^43,44^. Also, the suitable construction of the periodically poled lithium niobate layer can decrease the chance of exciting higher order non-linearities^6^. Our numerical calculations predict that for higher values of the initial pump power, the needed propagation length reduces and the final rate of squeezing increases. Therefore, higher rates of squeezing can be achieved using the proposed plasmonic waveguide at shorter propagation lengths (Fig. 8). Here, the log-scale is used for the horizontal axis of the figure.

From Fig. 8, it can be seen that the SH mode squeezes more at much shorter propagation length (  $\sim 2~{\text {mm}}$) in the proposed plasmonic waveguide. In contrast, the maximum of the SH squeezing rate for the traditional Ti:LNB waveguide needs propagation length of the order of $60~{\text {mm}}$ in this case. On the other hand, the squeezing rate of the pump mode for $\text {P}_{0}=~10{{ W}}$ in the plasmonic waveguide is nearly that of the traditional Ti:LNB waveguide. This rate of squeezing is achieved at just $\sim 2~{\text {mm}}$ of propagation length in our plasmonic waveguide which is much shorter than that of the traditional Ti:LNB waveguide (  $\sim 100~{\text {mm}}$).

## Conclusion

To sum up, a full numerical method to calculate the squeezing procedure for the pump and for the SH modes through SHG process in the presence of the loss mechanism has been proposed in this article. Unavoidable items like pump depletion, complex nonlinear coefficient and nonlinear phase mismatch have been included in the squeezing procedure of nonlinear waveguides. This novel method can be used in other types of squeezing which use nonlinear optical waveguides like optical parametric amplification and difference frequency generation. The final form of the achieved numerical formula is compact and simple. The results show that the loss mechanism limits the squeezing performance due to accumulated noises leading to de-squeezing of the SH mode and increasing the uncertainty of the related quadrature. By applying our proposed method to a well-designed nonlinear plasmonic waveguide, it is shown that the efficiency of the simultaneous generation of the squeezed states for the pump and SH modes can be well improved. This is due to much greater value of nonlinear coupling coefficient of the plasmonic waveguide compared to that of the traditional nonlinear Ti:LNB waveguide. The results show that, by using the proposed plasmonic waveguide and a high value of initial pump power of the order of 10*W*, it is possible to generate squeezed states for a propagation length of about ${2}\;{\text { mm}}$ which is much shorter than that of the traditional LNB optical waveguide being of the order of ${100}\;{\text {mm}}$. Results of this work suggest the generation of squeezed states using nonlinear plasmonic waveguides for super-low-noise optical communications and super-high sensitive interferometry.

## Data Availability

There is no additional dataset for this study. Because, all the data generated or analyzed during this study are included or referred in this published article. However, For the clarification and convenience of other authors and researchers, any needed datasets used and/or analyzed during the current study is available from the corresponding author on reasonable request.
